# Nepali Migrant Workers and the Need for Pre-departure Training on Mental Health: A Qualitative Study

**DOI:** 10.1007/s10903-019-00960-z

**Published:** 2019-12-18

**Authors:** Pramod R. Regmi, Nirmal Aryal, Edwin van Teijlingen, Padam Simkhada, Pratik Adhikary

**Affiliations:** 1 Faculty of Health and Social Sciences, Bournemouth University, Bournemouth House (B220), Bournemouth, BH1 3LH UK; 2 School of Human and Health Sciences, University of Huddersfield, Huddersfield, HD1 3DH UK; 3 Research Unit, Green Tara Trust, Kathmandu, Nepal

**Keywords:** Labour migrant, Mental health, Pre-departure, South Asia, Screening

## Abstract

Every year around 1000 Nepali migrant workers die abroad. Every one in three females and one in ten males commit suicide, reflecting a high mental health risk among Nepali migrant workers. This study aims to identify triggers of mental ill-health among Nepali migrant workers and their perceptions on the need of mental health components in the pre-departure orientation programme. We conducted five focus group discussions (FGD) and seven in-depth interviews with Nepali migrant workers and eight semi-structured interviews with stakeholders working for migrants. Participants were invited at Kathmandu’s international airport on return from abroad, at hotels or bus stations near the airport, through organisations working for migrants, and participants’ network. All FGD and interviews were conducted in Kathmandu and audio recorded, transcribed and translated into English. Data were analyzed thematically. High expectations from families back home, an unfair treatment at work, poor arrangements of accommodation, loneliness and poor social life abroad were frequently reported factors for poor mental health. Access to mental health services abroad by Nepali migrant was also poor. We found little on mental health in the pre-departure orientation. We need to improve our knowledge of mental health risks to provide better, more focused and more up-to-date pre-departure training to new migrant workers leaving Nepal.

## Introduction

Approximately 3.5 million Nepali are working abroad, primarily in Malaysia, the Gulf Cooperation Council (GCC) and India [1] and most are involved in high-risk unskilled jobs, men mainly on building sites, in factories and women mainly in domestic work. Currently, international migration is a livelihood strategy for many Nepali workers who send over US$6 billion back home every year, comprising 26.3% of Nepal’s gross domestic product (GDP) [2]. However, this income is often generated at a great personal cost to the workers. The literature highlights several health issues of migrants in GCC and Malaysia, such as accidents and injuries due to poor health and safety at work [3–5]. *The Foreign Employment Board (FEB) of Nepal* reports around 1000 deaths of Nepali migrant workers in the GCC and Malaysia every year and of these around 1 in 10 deaths in males and 1 in 3 deaths in females are due to suicide [1].

Mental health has attracted much attention globally following a strong statement of the World Health Organization (WHO) in 2005: “There is no health without mental health” [6]. The importance of mental health has been further recognized in the Sustainable Development Goals (SDGs), primarily in Goal 3′s target to promote mental health and wellbeing [7]. Studies have documented factors that trigger poor mental health among migrants. For example, migrants in Canada highlighted that racial discrimination is one of the important mental health risk factor for many immigrants [8–11], whilst immigrants in US experienced stress, sadness and anxiety [12]. Studies among labourers from GCC also document evidence that migrant workers are at higher risk of depression, anxiety and suicidal ideation [13–15].

The International Organization for Migration (IOM) considers a pre-departure orientation programme as an important tool to: (a) reduce the vulnerabilities of migrant workers; and; (b) enable them to maximize benefits from overseas employment [16]. In Nepal pre-departure orientation has been mandatory since 2004 to all aspiring migrant workers to promote their health and wellbeing. Currently, the orientation programme runs through 147 training centres registered with the Department of Foreign Employment. These centres are mostly situated in the capital Kathmandu. The training resources are developed by the FEB in coordination with different relevant stakeholders including the International Labour Organisation (ILO) and IOM. The trainers of these orientation programmes receive regular curriculum-based capacity building training from the FEB. The orientation programme is also monitored and evaluated by FEB. The training curriculum includes topics such as: occupational health and safety, Human Immunodeficiency Virus (HIV), communicable diseases, sexual and reproductive health (SRH), mental health, road traffic rules, labour laws, and lifestyle issues abroad. Mental health coverage in the pre-departure training curriculum is currently limited. At the moment, there is no mental health screening for Nepali migrant workers prior to their departure.

Despite a significant number of Nepali migrants working abroad, factors that affect their mental health and wellbeing are poorly studied. Similarly, migrants’ perceptions of the existing pre-departure training are not well documented. Against this background, this qualitative study aims to explore: (a) factors affecting mental health of migrants and utilisation of mental health care services abroad; and, (b) the perceptions of aspiring migrants about mental health related contents in pre-departure training curriculum.

## Methods and Materials

### Research Design and Participants

This qualitative study uses both Focus Group Discussions (FGDs) and interviews [17, 18]. In late 2017, we conducted four FGDs with returnee migrants (male-3 groups, female-1 group) and one separate FGD with aspiring migrants going abroad; with 6–8 participants in each group (characteristics of FGD participants are presented in Table 1). We defined returnee migrant as “Nepali migrant workers aged 18 years or above and who have worked abroad for at least 6 months as a labour migrant”. We also carried out seven in-depth interviews (Table 2) with the aspiring migrants who attended pre-departure trainings in Nepal.

**Table 1:** Characteristics of FGD participants

FGD groups	Age range (yrs)	Ethnicity	Destination countries	Occupation abroad	Years lived abroad
FGD 1 male	23–45	Chhetri-5 Terai Caste-1	Qatar-4 Saudi Arabia-2	Labourer-6	1.6–7
FGD 2 male	20–32	Terai Caste-6	Qatar-6	Labourer-5 Driver-1	1–7
FGD 3 male	25–41	Dalit-1 Chhetri-6	Malaysia-6 Saudi Arabia -1	Labourer-2 Factory worker -2 Other-2	1–3
FGD 4 female	22–53	Tamang-2 Magar-2 Brahmin-2	Oman-3 Malaysia-2 UAE-1	Domestic worker-3 Company work-2 Beautician-1	< 1–1.5
FGD 5 male (aspiring migrants)	21–28	Terai Caste-3 Magar-1 Chhetri-1 Dalit-1	Malaysia-4 Saudi Arabia-2	Labourer-2 Factory worker-4	Not applicable

**Table 2:** Characteristics of interviewees: in-depth/key informant interviews

Characteristics	Migrants (n = 7)	Key informants (n = 8)
Gender	Male-4, Female-3	Male-6, Female-2
Age range (years)	27–47	23–38
Occupation	Security, domestic worker, computer operator, labourer	Health care provider, trainer, recruitment agency, organization working on labour migrants
Duration of work	6 months to 12 years	1 to 5 years
Destination country	Dubai-1, Saudi-1, Qatar-2, Japan-1, Oman-2	Not relevant

In addition, eight semi-structured key informant interviews (KIIs) were conducted. Representatives from recruitment agencies, health care providers, trainers and governmental and non-governmental organisations (NGOs) working on migrants’ issues were included in these KIIs (see Table 2).

### Participant Recruitment

We recruited returnee migrants mainly at: (a) Tribhuvan International Airport (Nepal’s only international airport) during their return and through local hotels near airport/key bus stations where they usually stay for couple of days before departing to their homes; (b) organisations working for migrants; and, (c) migrants’ network, commonly known as snowball sampling [19]. For the KIIs, representatives from health facilities, governmental organisations (e.g., department of labour, FEB) and NGOs working on migrant health issues were approached.

### FGD and Interview Tools

Based on the available literature and discussions with key stakeholders, we drafted FGD and interview tools (copies available from first author on request). The FGD tool included issues on: (a) general life (e.g., facilities available in their accommodation) and working environment (e.g., workload, tea/meal-break during shift, bullying, timely salary, injury, compensation at work); (b) pre-departure training (e.g., training attendance before going abroad, usefulness of training [coping with work pressure, family issues, isolation, homesickness)]; and, (c) mental health issues abroad. Similarly, the key informant interviews with stakeholders included questions around challenges to include or implement mental health components in curriculum (e.g., lack of appreciation on mental health issues by aspiring migrant workers and policymakers, lack of trainers, poor literacy rate of aspiring migrant workers, time constrained, and competing priorities). The FGD guide was used in all FGDs as a starting point and the interview guide was adjusted for each KII depending on their background. Through a FGD with migrants, we piloted the FGD and interview tools before going live with the participants [20].

### Data Collection

FGDs and interviews were facilitated by an experienced qualitative researcher. They were audio recorded with the permission of the participants. Private venues (e.g., hotel rooms or NGO offices) were used for the FGDs whereas KIIs were conducted in participants’ offices or by telephone. FGDs, in-depth interviews and KIIs lasted approximately 1 h, 45 min and 30 min respectively.

### Data Organisation and Analysis

We transcribed the original recordings in Nepali and then the transcribed data/notes were translated into English. PRR and PA (both native Nepali speakers) independently reviewed the transcription and translation. Any disagreements were discussed in detail between the research team for appropriate translation. Each transcript had a cover note describing the interview, setting, how the discussion had established, any differences to other interviews, particular incidents, the environment and a reflection on the issues identified in the session. Any differences between the first and the second coder were discussed in the team until consensus was reached. The transcriptions were coded with the help of NVivo 11 (QSR International Pty Ltd, Australia) [21]. We performed a thematic analysis and relevant quotes are presented below to illustrate the key themes [22].

## Ethical Consideration

We received ethical approvals from Bournemouth University’s Research Ethics Committee (Ref: 16409) and the Nepal Health Research Council (Ref: 13/2017). Written informed consent [23] was obtained from all participants prior to the FGDs and interviews. Through an information sheet in Nepali, participants were provided with enough information about the study procedure, confidentiality, study purpose, risk and benefits to the participants, complaint procedure. Mobile phone recharge vouchers (worth less than US$ 2) were provided to each FGD and in-depth interview participants as a reward for their participation.

## Findings

We present our findings under three key themes: (1) factors affecting mental health and wellbeing; (2) perceptions on pre-departure orientation; and (3) access to and utilization of health-care services.

## Factors Affecting Mental Health and Wellbeing

During the FGDs, participants discussed several factors that affect their mental health and wellbeing. These are presented under five different sub-themes namely: (a) families as a source of poor mental health; (b) unfair treatment at work place; (c) poor arrangement of accommodation abroad; (d) poor social life abroad; and, (e) loneliness and insecurity. Each theme is discussed in turn below.

### Families as a Source of Poor Mental Health

Most participants reported that their families back home affected their mental health and wellbeing. There were two key issues highlighted by the FGD participants: (a) living far away from family; and (b) high expectations from family. The notion of not being able to help their families back home was frequently reported, for example, one female participant stated:

“I used to miss my children a lot. I always thought that because of poor economic condition I was there (abroad), leaving my small children in Nepal and at the most crucial time when they needed me. Despite of giving them love and education, I was there to earn. I was really struggling there for income, how to earn more in less time?… I really had a stressful life.” (FGD, returnee migrant, female)

Another participant refers to the pressure she felt having to work to make money for her family back in Nepal:

“…I had two small kids here in Nepal. …. At that time I could not leave the job because I had a responsibility of my family. I used to cry almost every day. I had even thought about suicide.” (FGD, returnee migrant, female)

Participants also argued that their families back home have high expectations of them, in terms of giving them presents and/or money, for example:

“Our family and relatives expect gift, goods and money from us saying ‘You have been to foreign for so long, you must have earned a lot, you didn’t give us anything’. This really causes mental stress on us.” (FGD, returnee migrant, male)

They stated that there is always a worry about the misuse of the money that migrants send back home which stresses them, for example:

“I worked hard [= abroad] and sent money for the family at home. There will be stress if they do not use the money in a proper way at home here in Nepal. In my experience, family related issues cause us mental tensions.” (FGD, returnee migrant, male)

### Unfair Treatment at Work

Many participants had experienced some kind of discriminatory behaviours from their supervisors or employers, co-workers or house owners (landlords). They believed that compared to other foreign workers Nepali workers face more problems. Such discriminatory behaviours in their work could worsen their mental wellbeing.

“…there is a huge discrimination between senior and junior. Most of the time they [= supervisor] complained about us and tried to find weakness from our work so that we could not get overtime hours. Even if I did a good job then also either by mixing low quality products or by making error themselves, they tended to reject our work in front of the main boss. Our supervisor was jealous if we worked well and earned more. I feared to talk with seniors or even with co-workers due to the possible consequences.” (FGD, returnee migrant, female)

“I planned to commit suicide several times when they [= work agent] misbehaved [= physical torture].” (In-depth interview, male)

Migrant workers perceived that they were not only discriminated against in their host country but they also experienced discrimination from the recruitment agencies in Nepal. Upon their arrival in a destination country, migrants experienced a different reality to the one they had been expecting. One FGD participant explained:

“We had to work in a different place than we were earlier told by the agent in Nepal. They promised indoor jobs [work inside the company] but when we arrived, we were assigned work in road construction.” (FGD, returnee migrant, male)

“Agents asked us to tell lie in the Kathmandu International Airport…I got less salary than the manpower agency promised in Nepal.” (FGD, returnee migrant, male)

There was a consensus among participants that discrimination they face at work triggered poor mental health. They further argued that migrants were unable to report these kinds of unfavourable work environments to their employers or agents in Nepal due to the job insecurity.

Most of our participants believed that their employers undervalued the work of migrant workers and paid poorly and irregularly, some were not paid at all by their employers. They believed that the irregular or low payments increased their day-to-day costs of living in the host countries and Nepal. Some had colleagues who committed suicide, as one migrant shared:

“One of my co-workers committed suicide as he did not get salary. What else he could do? It had been almost 10 months he worked but did not get salary, neither did the company allow him to get back to home.” (FGD, returnee migrant, male)

One of the key interviewees argued that there is a need for research into this issue:

“We heard about deaths of migrants every day. Heart diseases and suicides are said to be common. A study is necessary to know more about why this is happening with our brothers and sisters.” (Key informant interview)

Workload and work pressure were frequently linked with poor mental health, for example:

“We had to finish given task on time…there was always a tight schedule, therefore sometimes not possible to complete on time. Temperature was high and people were tired of working in high temperature and humidity. Senior manager put pressure on us to complete the tasks at all costs. In this situation, workers became very stressed.” (FGD, returnee migrant, male)

Domestic workers mentioned that families who employ them may treat them badly, for example:

“House owners misbehave and shout at us. It feels that we were bonded labours. Please, help those Nepali migrants who are at risk there. … It was our mistake because we went there without getting permission from Nepal.” (In-depth interview, female)

### Poor Arrangement of Accommodation Abroad

Most returnee migrants in the FGDs lived in poor accommodations with limited facilities. For instance, rooms were too small for the allocated number of people. Participants often mentioned lack of security, poor hygiene, such as dirty toilets and bathrooms. They felt that poor accommodation facilities as such resulted in poor mental health:

*“Labourers [*= *migrant workers] had to live in a very bad [*= dirty] place. Companies provided us the rooms or flat which were in a dire state. We had to live there like cattle. Sometimes we had to sleep on floor as mats or carpets were not provided. We felt very bad and our self-esteem was very low during that time.” (FGD, returnee migrant, male)

Interestingly, some participants argued that accommodation of Nepali migrant workers are generally poorer than the accommodation of migrants from other countries. One female returnee migrant shared:

“Accommodation for Vietnamese, Indonesian workers were well facilitated with high security, but for Nepali was very poor, in terms of both facilities and security. There was always danger of theft in our place. Once my mobile and money were stolen from my room. I always felt insecure and worried.” (FGD, returnee migrant, female)

### Poor Social Life Abroad

Our participants explained that they had a very limited social life. They unanimously agreed that their quality of life abroad was not good. If men had any time at all for entertainment or recreation, they always spent it by worrying about their work, life, families back home or their debt, hence, they did not live happily. In contrast, most female migrants worked as a domestic worker and as such had limited time for entertainment. They were often not given days off or any leisure time. The families that employed female migrant workers usually forced them to work for excessively long hours every day.

“I had a very bad time there… I never attended a social programme of the Nepali community because I was not allowed to participate. I was always given some work with tight deadlines. If I had attended any social programme, I could have met friends and could have shared my pain…I had almost gone mad.” (FGD, returnee migrant, male)

Interestingly, some returnee participants from Malaysia said that Nepali workers were engaged in multiple affairs or sex partners. They argued that multiple affairs put women at risks of dying in Malaysia. This not only impacted the victim or their family in Nepal, but also affected psychosocial wellbeing of other migrant workers. One of them exemplified:

“I wondered why Nepali workers were dying there [= in Malaysia], why people were posting messages of suicide, dead pictures on Facebook. Some of our friends also had multiple affairs. When they date, their boyfriend kills them if they find out [= about relationship with others]. …. When we hear these kinds of things, we feel really worried because local people may see us collectively as loose character women.” (FGD, returnee migrant, female)

### Loneliness and Insecurity

Loneliness was one of the factors triggering poor mental health in Nepali migrants. Participants argued that they were alone in a foreign country. Stress was generated due to being exposed to a new environment, new language or lack of confidence confined them within the premises of work or quarters, therefore, stressing them, for example, one returnee migrant shared:

“Loneliness is common after leaving home because we cannot speak with other people. We also don’t know where to go. But heavy workload, poorer than expected working conditions, and working full stop adds further stress.” (FGD, returnee migrant, male)

The heavy work load also restricted their opportunities to communicate with family back in Nepal,

“We always missed our family. Due to the workload, we could not talk with them whenever we liked… That made me feel sad.” (In-depth interview, returnee migrant)

Some argued that poor communication facilities with families had also impacted their mental health and wellbeing.

“There was no access to communications with families. We could communicate each other with family if we had access to phone. We did not have permission to use mobile phone. These are the causes of mental health problems among female migrants in the domestic work which brings suicidal thoughts.” (In-depth interview, female)

Some participants explained that there were feelings of insecurity and fear while living abroad which also impacted on their psychological wellbeing. They were less confident walking around the streets, shopping or making new friends abroad. Female participants highlighted that migrant women usually did not have any choice except working. They were physically abused, thus felt very insecure. They repeated that these unfavourable working environments are also sources of their stress and anxiety.

## Perceptions on Pre-departure Orientation

Most participants had positive feelings towards the pre-departure training. They agreed that such pre-departure orientation helped to understand many elements of migration such as immigration process, local weather, culture and language of receiving countries, rules and regulation at work and lifestyles abroad. When asked whether the training included components around health, participants mentioned that there were discussions around different health aspects. They explained:

“We were told that we should not use drugs [= recreational drugs] while staying abroad because we go there for work. If we adopt bad habits, it definitely affects us.” (FGD, aspiring migrant, male)

“There was a discussion about health, visa process and airport related procedure. We should be waiting when we reach there [= airport in destination country]. It was told that there is also provision of health check up in the destination country. We were told that we should work properly as assigned by employer.” (FGD, aspiring migrant, female)

However, participants thought that the pre-departure orientation should include detailed information about mental health, particularly about the causes of poor mental health and what possible mental health services migrant workers can use abroad. Most suggested that mental health is very important to reduce work-related stress and anxiety. Participants unanimously agreed that the training and orientation around mental health could help them to cope with emotional difficulties or reducing self-harm, for example:

“…because we go through tensions and anxiety while in abroad, training on mental health will really help us to reduce negative incidents…like suicide.” (FGD, returnee migrant, male)

The key informants acknowledged that health issues are discussed in the pre-departure orientation programme. However, they also agreed that the current orientation programme is not very inclusive and there are no comprehensive mental health components:

“There are several topics covered…such as legal and environment related things. It also includes health section which covers minimum 3 h sessions. HIV is also included there especially on how to prevent from HIV. Information about alcohol use is also there. I feel there is a little information about mental health but not enough.” (Key informant interview)

“I think the programme should be very focused on mental health. At the moment they just touch on different issues. Migrants may know some health issues due to the training but may not utilize the knowledge into practices.” (Key informant interview)

Another key informant highlighted the need of psychosocial components in the orientation that may help maintain family relationship back home.

“I think knowledge on psychosocial factors will help migrant workers to stay long time in foreign employment without stress. If there is no communication with parents and family, it might create family destruction related things. It really affects children and social structure.” (Key informant interview)

Some FGD participants questioned the quality of the orientation programme. They felt that it was conducted just for the sake of it. Similar views were reported by some of our key informants working in NGOs, as exemplified by this quote:

“Training is perceived as a formality. This is done as it is mandatory and just for formality. Reports show that contents are good but trainings are not conducted properly. Training contents are good but there are many problems in implementation.” (Key informant interview)

## Using General and Mental Health Services Abroad

Returnee participating migrants shared the mixed experiences around the use of health services abroad. They argued that the health care use abroad is influenced by the place where they lived, nature of the companies they worked for, the generosity of employers, income levels of individual migrant workers and local transportation facilities. They shared that generally larger companies are more supportive and assist workers by taking them to hospital for treatment and providing good facilities:

“It was easy to receive health service. Our company used to take the injured workers immediately to hospital.” (FGD, returnee migrants, male)

“They had issued an identity card… they verified the name and provided travel fare and also authorized leave. This was how company supported us during any problems.” (FGD, returnee migrant, male)

However, some participants (particular those working as domestic cleaners) argued that small companies do not offer any support to worker even if there are health issues. Participants said that migrants have to bear all associated cost and some are even send back home. One female domestic worker explained:

“They [= employer] did not take me to any hospital. My mind was not working. One day they told me to pack my bags, (oh…) I arranged all my things in one big bag and they sent me here [= Nepal].” (FGD, returnee migrant, female)

Most of our participants said that accessing to mental health information and services abroad by Nepali migrant is very elusive. Those who had received such services had also faced communication problems. There was also a fear among the participants due to the stigma attached to mental health; hence, Nepali migrants rarely disclose feelings to family members or peers. Most participants accepted that even if any mental health-related issues appear, they are reluctant to disclose it otherwise family or friends may isolate them thinking that they are insane:

“There are many things that affect our health and mental health. When we suffer from stress or other mental health issues, we can’t go to doctors because we cannot share them. Also, we fear that if it is known to the employer we may be forced out from the work.” (FGD, returnee migrant, male)

“One of my friends had mental problem after coming to Malaysia. We suggested to him to share with his family. I heard that he talked with his wife about this but he did not get support from her either. He then left work. I do not know where he is these days.” (FGD, returnee migrant, male)

## Discussion

To our knowledge, this is the first study to: (a) explore factors which affect mental health and wellbeing of Nepali migrants; and, (b) elicit migrants’ views on the mandatory pre-departure orientation programme. Our qualitative study found that the pre-departure orientation for Nepali migrant workers is generally commendable; however, there is a need for detailed sessions/discussion on mental health. Their key concern is contents (for instance, lacking adequate mental health components) and duration of the training is not as long as necessary. Participants often raised issues around the quality of the trainings. This signals that there is an urgent need to reassess, redesign and regularly monitor the quality of the training. This will help customize the content and the effective delivery of the orientation training.

Evidence suggests that training and development programs for migrants are becoming more widespread [24]. Migration is a stress-inducing phenomenon [25, 26] and migrant workers have to cope with psychologically stressful conditions, for example, lack of preparation and difficulties in adjusting to the new environment, which may lead to depression and anxiety [25, 27]. Cross-cultural training and orientation to migrant workers may enhance their adaptability to and familiarization with the host country environment [28]. As most Nepali labour migrant workers are from low educational background and may not be fully aware of potential health risks (including mental health), an orientation programme may help to minimize the risks through: (a) offering sessions to improve language skills they communicate at work; (b) providing adequate information about health risk such as ‘what causes poor mental health’ and offer information about the service points they may visit to access information and health care; (c) encourage to enroll for health insurance; and, (d) conducting awareness programme focusing on female migrant workers to reduce physical abuse including violence.

In our study, migrants reported several factors that may trigger poor mental health. Particularly, high expectation from family back home, poor living arrangements and working environment, discrimination at work, poor social life and loneliness in abroad are the frequently reported factors for poor mental health. These were often linked with low self-esteem and poor quality of life abroad. We found that many Nepali migrants experience isolation or discriminatory behaviours at both their work place and at the quarters or community they live. These social isolation and discrimination at work may worsen their mental health. For example, issues of irregular payments, not only impeded timely support to their family back home but could also trigger self-harm behaviours. A few studies among migrant workers in the Middle East report that suicide rates are higher among immigrants compared to local population [13–15]. Worryingly, migrants are unable to share their problems to relevant stakeholders due to the job insecurity and cultural stigma about mental illness.

Nepal currently does not collect national data on the burden of mental illness. Neither does it have any in-depth studies to explore mental health and wellbeing of Nepali migrants. Thus, we are not able to directly compare our study result with other Nepali populations. Evidence on mental health issues among migrant workers is scarce globally. However, some studies indicate a significant mental health risk in this population. A review on mental health problems among migrant workers in GCC reported significant risks, mainly adjustment disorder, mood disorder, psychosis and suicide [29]. Work-related circumstances could trigger mental health problems in migrant workers such as excessively long working hours, no days off, delayed or non-payment of wages, physical and sexual abuse [30].

Another important aspect triggering poor mental health among migrant workers is loneliness. Feeling lonely abroad may not only affect their mental health and wellbeing, but also increases risk taking behaviours. A review reported that sexual risk taking including visiting female sex workers is common among male Nepali migrant workers [31].

We found that the uptake of health care (including mental health) by migrants abroad was poor. Stigma attached to mental health further discouraged migrants from seeking such services. They did not discuss their mental health with their family due to the fear of stigma and ostracization. We argue that promoting awareness on the impact of some cultural attitudes/practices will: (a) help encourage a better uptake of mental health services; and (b) make discussing mental health issues easier in both the destination and host countries.

We acknowledge a couple of limitations of this study. First, being a qualitative study, it is difficult to establish an association and cause-effect relationship of identified mental health risk with migrants. Future cross-sectional surveys and longitudinal study may provide such information with high certainty. Secondly, we selected our participants purposively from Kathmandu; our findings therefore may not represent the voices of returnee migrants from other parts of Nepal who might have travelled to their destination countries from airports in northern India. Thirdly, as mental health is considered a taboo subject, some participants might have underreported experiences and perceptions.

## Conclusion

There are many factors such as high expectations from families back home, an unfair treatment at work, poor working and living arrangements, discrimination at work, social isolation abroad which may trigger poor mental health of Nepali migrants. The pre-departure orientation programme for aspiring migrants is not comprehensive enough and there is an urgent need for a greater mental health component in the curriculum. The uptake of health services including mental health care is poor. There is a need to improve our knowledge of risk factors for poor mental health and suicide (ideation). We need such knowledge to improve both mental health screening of migrant workers prior to departure, add more appropriate mental health components to the pre-departure orientation curriculum and promote better support activities in destination countries. We argue that there is an urgent need to provide better, more focused and more up-to-date pre-travel training to new migrant workers leaving Nepal.
